# Supplementary material for: Ion Transport Modulators Differentially Modulate Inflammatory Responses in THP-1-Derived Macrophages
Source: J Immunol Res. 2021 Apr 7;2021:8832586. doi: 10.1155/2021/8832586 (PMC8049803; doi:10.1155/2021/8832586)
Supplement: Supplementary Materials — Figure S1: mean (+SD) viability of THP-1-derived macrophages following exposure to different concentrations of test drugs. [file 8832586.f1.pdf]

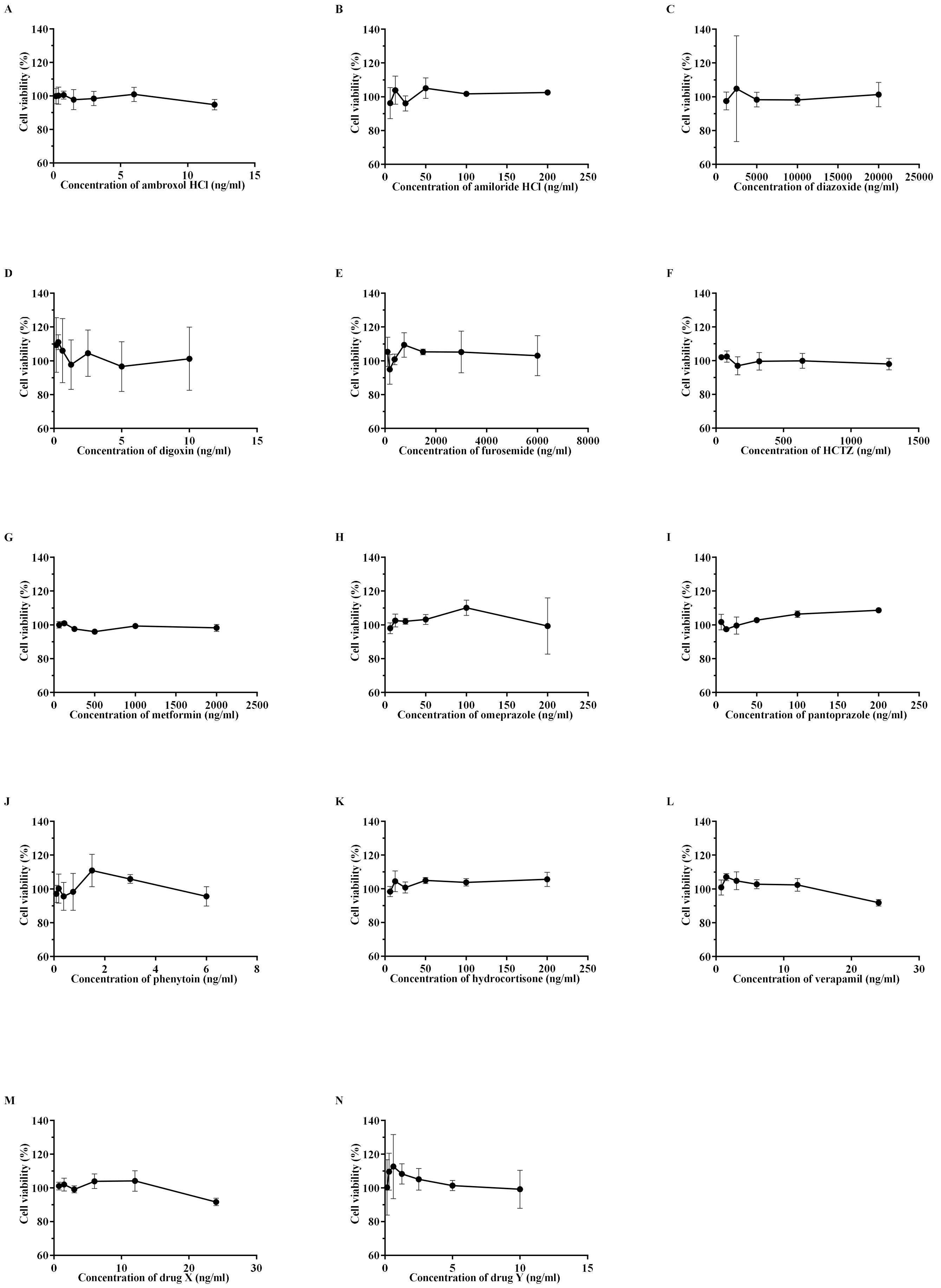

Figure S1: Mean (+SD) viability of THP-1 derived macrophages following exposure to different concentrations of test drugs. HCTZ, hydrochlorothiazide. Reprinted from "Ion transport modulators as antimycobacterial agents," by SC Mitini-Nkhoma et al, 2020, Tuberculosis Research and Treatment, vol. 2020, https://doi.org/10.1155/2020/3767915.
